# Prevalence and severity of ectopic eruption of first permanent molars in 5–12-year-Old children attending a tertiary dental hospital in Dubai, United Arab Emirates

**DOI:** 10.3389/fdmed.2026.1927212

**Published:** 2026-08-26

**Authors:** Ali Mohammed Akbar, Mawlood Kowash, Iyad Hussein, Anas Salami, Amar Khamis, Manal Al Halabi

**Affiliations:** 1 Ministry of Health, Kuwait City, Kuwait; 2 Hamdan Bin Mohammed College of Dental Medicine, Mohammed Bin Rashid University of Medicine and Health Sciences (MBRU), Dubai Health, Dubai, United Arab Emirates

**Keywords:** ectopic eruption, first permanent molar, malocclusion, pediatric dentistry, prevalence, severity

## Abstract

**Background:**

*Ectopic eruption* (EE) of first permanent molars (FPMs) is a developmental disturbance that can lead to premature loss of the second primary molar (SPM), space loss, and malocclusion if not detected early.

**Objective:**

This retrospective, record-based cross-sectional review determined the prevalence and severity of FPM ectopic eruption among children in this Dubai tertiary-care cohort and compared findings with international data.

**Methods:**

Dental records and radiographs from 962 children aged 5–12 years who attended a tertiary dental hospital in Dubai between 2020 and 2024 were reviewed. Ectopic FPMs were identified radiographically and classified into four grades according to SPM root resorption. Data were analyzed using chi-square and Fisher's exact tests, with statistical significance set at *α* = 0.05.

**Results:**

Thirty-nine children (4.1%) had at least one ectopic FPM, corresponding to 61 affected teeth and a per-tooth prevalence of 1.6%. Ectopic eruption was more frequent in the maxilla than in the mandible, and patient-level bilateral involvement occurred predominantly in the maxilla. Most cases were mild, with severe cases being uncommon. No significant associations were found with sex, age group, nationality, or health status.

**Conclusion:**

In this hospital-based Dubai cohort assessed across a broad 5–12-year age range using available diagnostic radiographs, FPM ectopic eruption showed a mid-range prevalence with a maxillary predilection. Early radiographic assessment and timely interceptive intervention remain important to prevent complications and support optimal occlusal development.

## Introduction

Ectopic eruption (EE) is a disturbance of tooth eruption in which a developing tooth deviates from its normal eruption path, resulting in abnormal positioning or impaction. EE of the first permanent molar (FPM) occurs when the erupting FPM becomes lodged beneath the distal undercut of the second primary molar (SPM) and fails to reach the occlusal plane in its expected position (1–3). The condition is usually identified radiographically or following delayed or altered FPM eruption, often with evidence of pathological resorption of the adjacent SPM roots (1, 4). If not detected and managed early, EE of the FPM can result in progressive SPM root resorption and premature loss, loss of arch length and space for the second premolar, mesial drift/tipping of the FPM, and subsequent malocclusion (1–3, 5, 6). Its etiology is multifactorial and may include a mesially angulated eruption path, increased FPM crown size, and insufficient posterior arch space related to inadequate arch length or jaw growth (2, 7). Associations with other dental anomalies, including palatally displaced canines, and a possible familial component have also been described (2). Early diagnosis is therefore important, particularly for cases unlikely to self-correct. FPM EE may be reversible, with spontaneous correction often occurring by approximately 7 years of age, or irreversible, when the FPM remains locked beneath the SPM and requires active intervention (1, 2). Management depends on the degree of SPM root resorption and the child's dental development, ranging from orthodontic separators and distalizing appliances to extraction of the SPM in advanced cases (2, 5, 6).

Reported prevalence rates of FPM EE vary across populations and study settings. Estimates range from 0.75% in Thailand (7) to 13.9% in a clinical sample from Romania (8), with intermediate rates reported in Spain (8.7%) (9), Iran (10.2%) (10), Turkey (2.65%) (11), Saudi Arabia (2.2%) (12), and Kuwait (5.5%) (13). These differences are likely influenced by sample characteristics, diagnostic criteria, radiographic methods, and whether participants were drawn from community or referral-based clinical populations (2). Across studies, maxillary FPMs are more frequently affected than mandibular FPMs, and bilateral cases are commonly reported in the maxilla (7, 9, 11, 12, 14). Evidence regarding sex and side predilection remains inconsistent, with some studies reporting a higher frequency in boys and others finding no significant difference (7, 11, 12, 14, 15). Most reports indicate that mild or potentially reversible presentations predominate, whereas severe irreversible cases are less common (2, 6).

The present study aimed to determine the prevalence and severity of FPM ectopic eruption in 5–12-year-old children attending a tertiary dental hospital in Dubai, UAE, and to compare these findings with published data from other populations. In addition, the study examined differences in FPM EE occurrence and severity by sex, age group, nationality, arch, and side.

## Materials and methods

This retrospective, record-based cross-sectional review is reported in accordance with the STROBE guidelines for observational studies (16). Institutional ethical approval was obtained from the Research Ethics Review Committee at MBRU (Reference # MBRU-IRB-2020-037). We identified all children aged 5–12 years who attended the pediatric dentistry clinic at Dubai Dental Hospital (DDH) from June 1, 2020, through February 29, 2024. From these records, all eligible patient files that included clinical notes and radiographs were collected and reviewed.

### Sample size calculation

Given the lack of prior local data, *a priori* sample size estimation was conducted to ensure sufficient precision for prevalence assessment. Anticipating an FPM EE prevalence of approximately 5% and using a 95% confidence level with ±2% absolute precision, a minimum of approximately 457 children was required. This calculation was based on a single-proportion formula with *Z* = 1.96, *p* = 0.05, *q* = 0.95, and *d* = 0.02. The final sample of 962 children exceeded the required size and improved the precision of the overall prevalence estimate, providing a margin of error of approximately ±1.4%; however, subgroup analyses should be interpreted cautiously given the limited number of EE cases.

#### Inclusion criteria

Complete digital dental records for patients aged 5–12 years with at least one diagnostic radiograph (bitewing, periapical, or panoramic) clearly visualizing all erupted first permanent molars and the adjacent second primary molars. Both spontaneously corrected (“jumped”) and impacted (“hold”) types of FPM EE were included.

#### Exclusion criteria

**Figure 1:**
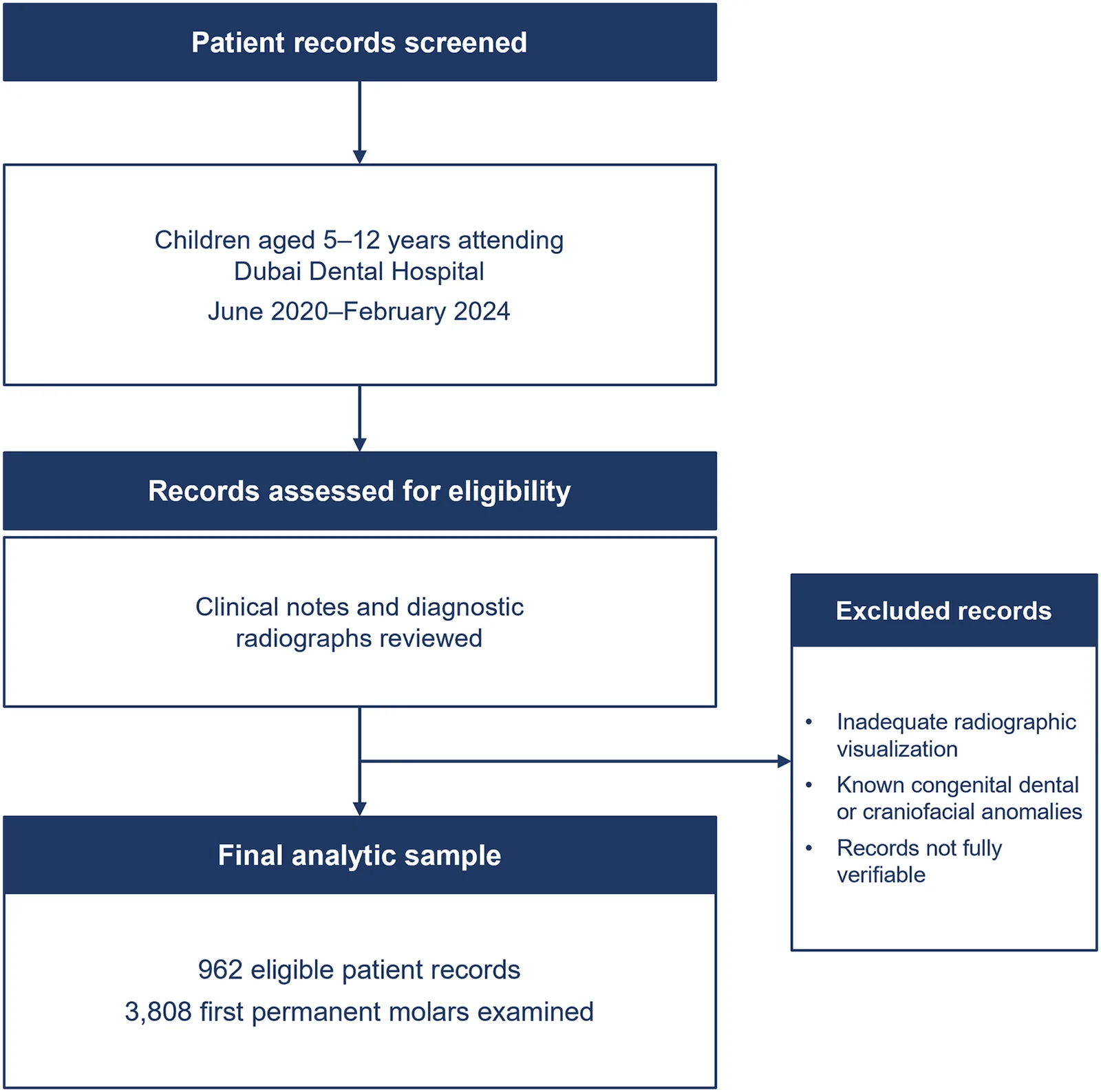
Flowchart illustrating the screening and inclusion of patient records in the study.

Records lacking adequate radiographic imaging of the relevant teeth; records of children with known congenital dental or craniofacial anomalies (e.g., cleft lip and palate); and any records that could not be fully verified. Children with medical conditions and/or special healthcare needs were not excluded unless they also had a concurrent craniofacial or dental anomaly. Figure 1 shows a flowchart of the screening and inclusion process for patient selection.

Prior to data collection, examiner calibration was performed. Two examiners (M.A.H. and A.M.A.) independently evaluated the radiographs to identify ectopic eruption and assign severity grades. Each examiner assessed the calibration set twice on two occasions, two weeks apart (5 bitewing, 5 periapical, 5 panoramic). Cohen's kappa coefficients were calculated to assess intra-examiner and inter-examiner agreement, yielding values of 0.75–0.81 (all *p* < 0.05), indicating substantial reliability. For the full dataset, any uncertain or discordant cases were re-evaluated by both examiners, and consensus was reached through joint side-by-side review of the radiographs, discussion with reference to the predefined EE definition and Hennessy-based grading criteria, and agreement on the final classification and severity grade. A pilot test on 10 randomly chosen patient records was also conducted to refine the data collection form before proceeding with full data extraction.

For each patient meeting the inclusion criteria, demographic data [age in years, sex, nationality (Emirati vs. non-Emirati), and general health status] were recorded. All available radiographs in each patient's file (via the Dental4Windows^TM^ digital imaging system) were examined. Children were classified as having ectopic eruption if EE was identified on any available radiograph during the review period. When serial radiographs were available for the same tooth, the highest observed severity grade was recorded. *Ectopic eruption* of an FPM was defined as an abnormally mesial eruption path that results in the FPM being wedged beneath the distal portion of the SPM, with or without associated resorption of the SPM. Each ectopic FPM was graded for severity of SPM root resorption using a four-tier radiographic scale adapted from Hennessy et al. (5). Because severity grading systems vary across studies, we selected this published framework to provide a clear, reproducible approach and to facilitate comparison with studies using similar definitions. The grades were defined as follows: **Grade 1 (Mild)**—limited resorption into cementum or minimal dentin penetration; **Grade 2 (Moderate)**—resorption into dentin but no pulp exposure; **Grade 3 (Severe)**—significant resorption of the SPM's distal root leading to pulp exposure; **Grade 4 (Very severe)**—extensive resorption involving the SPM's mesial root. Unilateral and bilateral EE were classified at the patient level within each arch, according to whether one or both sides of the arch were affected in the same child.

Unit of analysis: Prevalence was reported at two levels: (i) patient-level (children with ≥1 ectopic FPM) and (ii) tooth-level (ectopic FPMs among all examined FPMs). Because teeth within the same child are not statistically independent, associations with demographic and patient characteristics were tested at the patient level. In addition, tooth-level comparisons by arch/side and severity distribution were explored using chi-square or Fisher's exact tests; these inferential results are interpreted cautiously because they do not explicitly model clustering of teeth within children.

## Results

### Study sample characteristics

A total of 962 patient records met the inclusion criteria. The mean age was 7.8 ± 2.1 years. Ninety-six children (10.0%) were classified as children with medical conditions and/or special healthcare needs, including asthma, autism, congenital heart disease, or thalassemia. The demographic characteristics of the study sample are presented in Table 1.

**Table 1:** Demographic characteristics of the study participants.

Characteristic	*n* (%)
Sex	
Male	485 (50.4)
Female	477 (49.6)
Nationality	
Emirati	317 (33.0)
Non-Emirati	645 (67.0)
Health status	
Healthy	866 (90.0)
Children with medical conditions and/or special healthcare needs	96 (10.0)
Age	
5–8 years	579 (60.2)
9–12 years	383 (39.8)

### Prevalence of FPM ectopic eruption

Of the 962 children examined, 39 (4.1%) had at least one ectopically erupting FPM (patient-level prevalence). Overall, 61 ectopic FPMs were identified among 3,808 examined FPMs, corresponding to a per-tooth prevalence of approximately 1.6%.

The prevalence of first permanent molar ectopic eruption by arch and tooth position, including the number and percentage of FPMs affected in each quadrant and arch, is presented in Table 2.

**Table 2:** Distribution and severity of ectopic eruption by tooth position.

Tooth	Upper right first permanent molar (Tooth #16)	Upper left first permanent molar (Tooth #26)	Lower left first permanent molar (Tooth #36)	Lower right first permanent molar (Tooth #46)
Severity grade, *n* (%)	*n* (%)	*n* (%)	*n* (%)	*n* (%)
Grade 1	7 (0.7)	7 (0.7)	8 (0.8)	7 (0.7)
Grade 2	5 (0.5)	7 (0.7)	2 (0.2)	1 (0.1)
Grade 3	5 (0.5)	4 (0.4)	1 (0.1)	1 (0.1)
Grade 4	4 (0.4)	1 (0.1)	0 (0.0)	1 (0.1)
Total FPM with EE	21 (2.2)	19 (2.0)	11 (1.2)	10 (1.1)
Total FPM without EE	929 (97.8)	937 (98.0)	939 (98.8)	942 (98.9)

FPM, first permanent molar; EE, ectopic eruption.

Most ectopic FPMs occurred in the maxillary arch (40/61 affected FPMs, 65.6%). This corresponded to 2.1% of all maxillary FPMs examined, compared with 1.1% of mandibular FPMs. The difference was statistically significant using Fisher's exact test (*p* = 0.02), as shown in Table 4.

At the patient level, bilateral EE was also more frequent in the maxillary arch than in the mandibular arch. In this sample, bilateral EE was observed in 12 children with maxillary involvement (1.3% of all children examined) and 5 children with mandibular involvement (0.5% of all children examined); this difference was statistically significant (*p* = 0.013; Table 3).

**Table 3:** Patient-level distribution of unilateral and bilateral ectopic eruption by arch among children with ectopic eruption.

Ectopic eruption laterality	Maxillary involvement, *n* (%)	Mandibular involvement, *n* (%)	*p*-value Fisher's exact test
Unilateral	16 (1.7)	11 (1.1)	0.912
Bilateral	12 (1.3)	5 (0.5)	0.013*

FPM, first permanent molar; EE, ectopic eruption. Values are presented as the number of children and the percentage of all children examined. Unilateral and bilateral status was classified at the patient level within each arch. The reported *p*-values compare the distribution of unilateral and bilateral involvement between maxillary and mandibular arches using Fisher's exact test.

* Statistically significant *p*-value.

### Severity of ectopic eruption

Grade 1 was the most frequent single category; Grades 1–2 together accounted for most cases (Table 4). Moderate to very severe resorption (Grades 2–4) was recorded in 15 of 40 ectopic maxillary FPMs (0.8% of all maxillary FPMs examined) and in 6 of 21 ectopic mandibular FPMs (0.3% of all mandibular FPMs). Fisher's exact test showed no statistically significant difference in severity distribution between the maxillary and mandibular arches (*p* = 0.13).

**Table 4:** Arch-level prevalence and severity distribution of ectopic eruption among examined first permanent molars.

Tooth	Maxillary FPMs	Mandibular FPMs	*p*-value Fisher's exact test
Total number of teeth examined	1,906	1,902	
Total number of teeth with EE	40 (2.1)	21 (1.1)	0.02*
Total number of teeth without EE	1,866 (97.9)	1,881 (98.9)	—
Grade 1	14 (0.7)	15 (0.8)	—
Grade 2	12 (0.6)	3 (0.2)	—
Grade 3	9 (0.5)	2 (0.1)	—
Grade 4	5 (0.3)	1 (0.1)	—
Grades 1–2	26 (1.4)	18 (0.9)	0.13
Grades 3–4	14 (0.7)	3 (0.2)	

FPM, first permanent molar; EE, ectopic eruption. Values are presented as *n* (%). Percentages are calculated using the arch-specific number of examined FPMs as the denominator.

* Statistically significant *p*-value. *p*-values were calculated using Fisher's exact test with **teeth as the unit of analysis** and should be interpreted cautiously because they do not explicitly account for clustering of teeth within the same child.

Furthermore, there was no statistically significant difference in overall EE prevalence between the right and left sides (1.6% each side, *p* > 0.4).

No statistically significant differences in EE prevalence were observed by sex, age group, nationality, or health status (all *p* > 0.05), as shown in Table 5.

**Table 5:** Occurrence of FPM ectopic eruption by patient characteristics and associated *p*-values.

Study sample	Patients without EE	Patients with EE	*p*-value Fisher's exact test
Male	463 (95.5)	22 (4.5)	0.615
Female	460 (96.4)	17 (3.6)	
Nationality			
Emirati	303 (95.6)	14 (4.4)	0.722
Non-Emirati	620 (96.1)	25 (3.9)	
Health Status			
Healthy	834 (96.3)	32 (3.7)	0.183
Children with medical conditions and/or special healthcare needs	89 (92.7)	7 (7.3)	
Age			
5–8 years	560 (96.7)	19 (3.3)	0.154
9–12 years	363 (94.7)	20 (5.2)	

Values are presented as *n* (%). *p*-values were calculated using Fisher's exact test.

**Figure 2:**
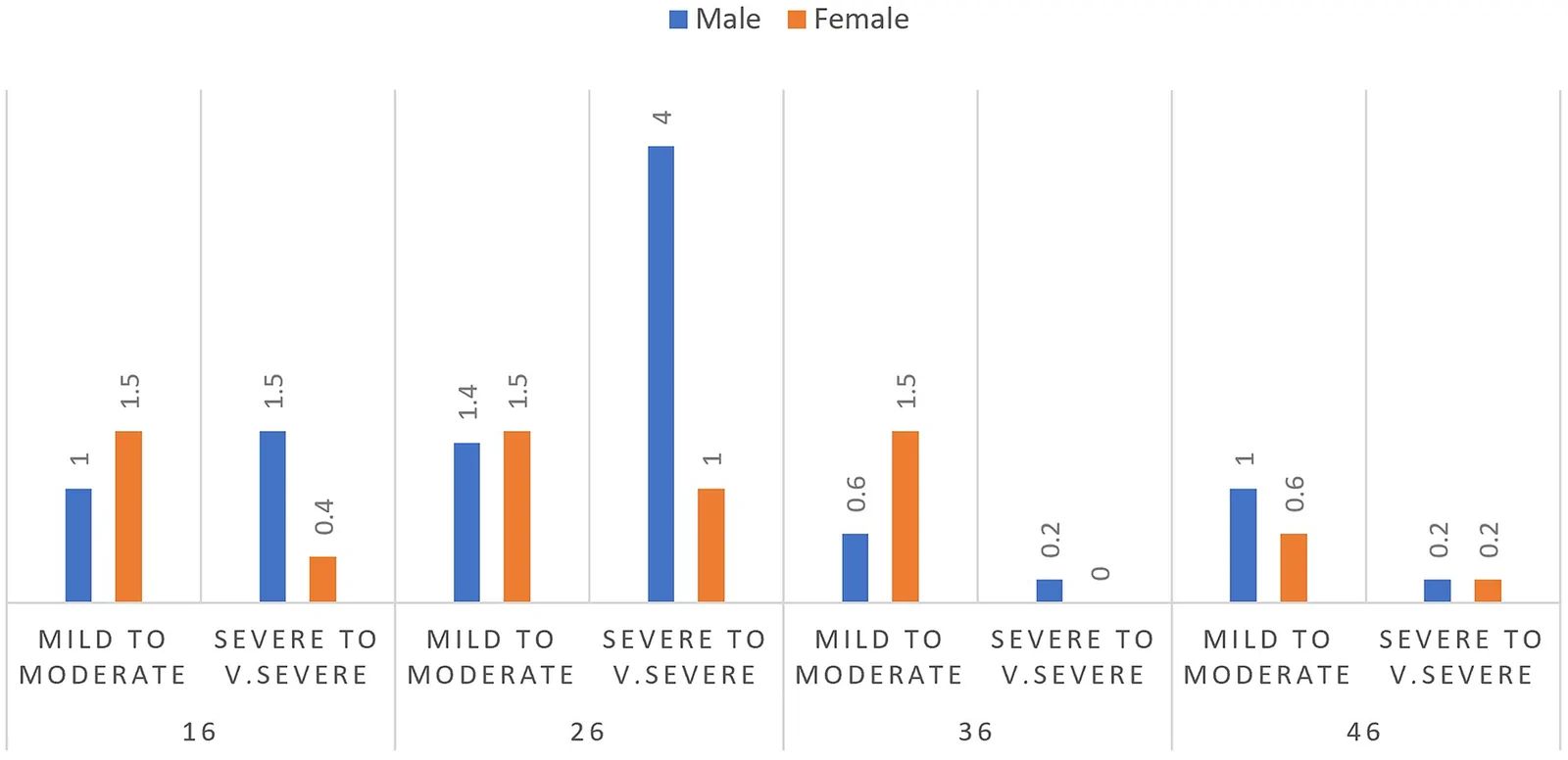
Comparison of the severity of ectopic eruption between male and female patients in all first permanent molars (16,26,23,46).

A non-significant trend toward greater severity was observed among female patients: 23.5% of ectopic FPMs in girls were classified as severe or very severe (Grades 3–4), compared with 12.5% in boys (*p* = 0.067). The comparison of EE severity by sex is presented in Figure 2.

## Discussion

This study provides baseline data on ectopic eruption of first permanent molars in this Dubai tertiary-care cohort. The hospital-based prevalence was 4.1% per child. This mid-range estimate likely reflects case ascertainment in our setting: we included both arches, applied radiographic case definitions across a broad 5–12-year age band, and relied on available bitewing/periapical/panoramic images rather than panoramic screening alone. Accordingly, prevalence was lower than higher estimates reported in Spain (8.7%) (9), Iran (10.2%) (10), and Romania (13.9%) (8), but higher than lower estimates from Riyadh (2.2%) (12) and Turkey (2.65%) (11), where narrower age windows, differing diagnostic frameworks, and/or imaging availability may capture fewer borderline cases (2).

The predominance of maxillary FPM involvement observed here mirrors a recurring pattern in the literature (7, 11, 12), and may reflect both **biologic** and **ascertainment** factors. Biologically, maxillary FPMs have been described as exhibiting a more mesially angulated eruption path and greater susceptibility to being locked beneath the distal contour of the SPM when posterior maxillary arch length is limited (2). Methodologically, studies that restrict case definitions to the maxilla or rely on radiograph types that better capture maxillary molar–SPM relationships can also report a stronger maxillary predominance (6, 9). At the patient level, bilateral EE was also more common in the maxilla, in agreement with earlier reports (9, 14).

While the maxillary–mandibular difference reached statistical significance in tooth-level analyses, its clinical meaning is best interpreted in absolute terms. In this cohort, the per-tooth prevalence was 2.1% in the maxilla vs. 1.1% in the mandible (an absolute difference of ∼1 percentage point, although approximately a two-fold relative difference). Clinically, this pattern does not imply that mandibular EE is rare or negligible; rather, it supports a higher index of suspicion for maxillary FPMs during early mixed dentition screening, consistent with other radiographic cohorts reporting a maxillary predominance while still documenting mandibular involvement (11, 12).

The sex distribution of FPM EE in this cohort was nearly equal (4.5% in boys vs. 3.6% in girls; *p* > 0.2), consistent with studies reporting no strong sex predilection (14). Although some studies have reported higher prevalence in boys (7, 11, 12), and a recent Turkish study reported a higher frequency in girls (17), our findings do not support a statistically significant sex-related difference in occurrence. Further population-based studies may help clarify whether sex-related differences in prevalence or severity are consistent across populations.

With respect to severity, most FPM EEs in this study were mild, consistent with reports that many cases self-correct or cause limited resorption when identified early (1, 2, 6). In clinical terms, Grade 1 reflects minimal resorption and is more likely to represent potentially reversible cases that may be monitored with short-interval review, whereas Grade 2 indicates dentin involvement without pulp exposure and typically warrants closer monitoring and early interceptive measures to prevent progression (2, 5, 6). Grades 3–4 represent advanced resorption (including pulp exposure and/or extensive root involvement) and are more consistent with irreversible impaction, where active intervention is often required to prevent premature SPM loss, space loss, and subsequent occlusal complications (2, 5, 6). Although moderate to severe resorption was numerically more frequent in the maxilla, the difference in severity distribution between arches was not statistically significant, therefore, it should be interpreted cautiously. Nevertheless, severe irreversible cases remain clinically important because they may lead to progressive SPM root resorption, premature loss of primary molars, and loss of arch space for the succeeding premolar. Management should be guided by the severity of impaction, SPM root resorption, and the child's stage of dental development, with options including separators, spring appliances, distalization, or SPM extraction in advanced cases (2, 5, 6).

Although many Grade 1 presentations may self-correct, ectopic FPM eruption is clinically important because progression can be insidious: ongoing impaction against the distal aspect of the SPM may lead to worsening root resorption, premature SPM loss, and subsequent space loss with downstream malocclusion if diagnosis is delayed (1, 2, 5, 6). From a preventive perspective, the principal value of early identification is not simply documenting prevalence, but triaging cases by severity and likelihood of self-correction, distinguishing mild, potentially reversible cases appropriate for short-interval review from Grades 2–4 cases that warrant timely interceptive management to prevent avoidable sequelae (2, 5, 6).

These findings support a structured approach during the early mixed dentition (approximately 6–8 years). Clinicians should assess the FPM eruption path and the SPM distal contour (reviewing available bitewing/periapical images or obtaining radiographs when clinically indicated), prioritize careful evaluation of the maxillary arch while still examining both arches, and check the contralateral maxillary FPM when unilateral EE is detected (9, 14). Mild Grade 1 cases may be monitored with short-interval review, whereas Grades 2–4 or lack of eruption progress should prompt timely interceptive measures (e.g., separators or distalizing approaches) and, when premature SPM loss occurs, consideration of space maintenance/regaining to minimize later space-management challenges (2, 5, 6).

Although ectopic eruption of the FPM primarily affects the adjacent SPM, more advanced presentations—particularly those associated with premature SPM loss and mesial drift/tipping of the FPM—may reduce posterior arch length and alter molar angulation, which could potentially influence the eruption path or alignment of the second permanent molar later in the mixed dentition (1, 2, 5, 6). Any implications for third molar position would be indirect and would occur much later, through cumulative changes in posterior space rather than through a direct effect of early ectopic eruption. Given our cross-sectional, radiograph-based design and the 5–12-year age range, eruption outcomes of second and third permanent molars could not be evaluated and should be explored in longitudinal follow-up studies (1, 2, 5, 6).

In this radiograph-based study, we did not perform standardized longitudinal assessment of occlusal contacts; nevertheless, ectopic eruption—particularly when unilateral and/or associated with premature SPM loss—may contribute to arch asymmetry and occlusal imbalance. Progressive mesial drift and tipping of the FPM together with localized space loss can predispose to midline discrepancy, crowding, and developing occlusal interferences, which in some children may manifest clinically as an occlusal shift during function. These considerations reinforce the need for clinicians to evaluate not only eruption path and resorption severity but also emerging molar relationships and occlusal contacts during follow-up, especially in Grades 2–4 cases where interceptive treatment and space management may reduce downstream occlusal disturbance (1, 2, 5, 6).

Given our cross-sectional, radiograph-based design, we cannot formally predict malocclusion outcomes; however, higher-severity presentations (Grades 3–4) may reasonably be considered higher risk for subsequent space loss and malocclusion due to their association with advanced SPM root resorption and potential premature SPM loss (1, 2, 5, 6).

## Limitations

This study has several important limitations. First, the retrospective, record-based design is inherently subject to incomplete documentation and limits control over exposure/outcome measurement and potential confounding. Second, case identification relied on radiographic assessment of existing images rather than standardized prospective clinical examinations; consequently, undiagnosed or undocumented cases may have been missed, and the estimated prevalence may be influenced by radiograph availability and indication. Third, because the dataset was cross-sectional and lacked longitudinal follow-up, we could not determine clinical outcomes over time (e.g., self-correction vs. persistence), quantify the progression of resorption, or evaluate treatment effectiveness.

Potential measurement and ascertainment bias is also relevant: variations in radiographic angulation, image quality, and interpretation can affect detection and severity grading, even with calibration, and the use of the highest recorded grade on serial images may over-represent peak severity in some children. In addition, heterogeneity in diagnostic and severity grading criteria across published studies may limit direct comparability of severity distributions between countries and study settings. Tooth-level inferential comparisons were exploratory and not adjusted for within-child correlation using clustered or multilevel models. As this was a single-center, tertiary-care sample, generalizability to the broader pediatric population may be limited, and prevalence estimates should be interpreted with caution. Broad inclusion criteria were used to maximize sample size and included both healthy children and children with medical conditions and/or special healthcare needs; however, subtle subgroup differences may not have been detected. Although the larger final sample improved the precision of the overall prevalence estimate, subgroup analyses should be interpreted with caution, given the limited number of EE cases. Future multi-center studies with larger pooled samples would increase the number of EE cases and improve power for subgroup and severity-based comparisons. Age was recorded in completed years rather than exact months, which may have introduced minor imprecision in determining the timing of EE identification. Finally, children with known dental or craniofacial anomalies were excluded to reduce confounding, so associations between FPM EE and these anomalies could not be evaluated.

## Conclusion

- **Prevalence:** The prevalence of ectopic eruption of first permanent molars in this Dubai tertiary-care cohort was 4.1% per child and 1.6% per FPM tooth.
- **Arch predilection:** Ectopic eruption of FPMs was significantly more frequent in the maxilla than in the mandible.
- **Severity:** Most ectopic FPMs showed lower-grade SPM root resorption. Severe to very severe SPM root resorption (Grades 3–4) was numerically more frequent in maxillary cases, but this difference was not statistically significant.
- **Bilateral cases:** At the patient level, bilateral EE was significantly more frequent in the maxilla than in the mandible, with bilateral involvement observed in 12 children with maxillary involvement (1.3% of all children examined) and 5 children with mandibular involvement (0.5% of all children examined).

### Clinical implications

Radiographic assessment around the expected period of FPM eruption is recommended to support early detection, guide timely interceptive treatment, and reduce the risk of premature SPM loss, subsequent space-management challenges, and downstream malocclusion.

## Data Availability

The raw data supporting the conclusions of this article will be made available by the authors, without undue reservation.
